# Evaluation of Cardiac Mitochondrial Function by a Nuclear Imaging Technique using Technetium-99m-MIBI Uptake Kinetics

**DOI:** 10.7508/aojnmb.2013.01.008

**Published:** 2013

**Authors:** Shinro Matsuo, Kenichi Nakajima, Seigo Kinuya

**Affiliations:** Department of Nuclear Medicine, Kanazawa University, Kanazawa, Japan

**Keywords:** mitochondria, ^99m^Tc-MIBI, ischemic heart disease, heart failure, cardiomyopathy

## Abstract

Mitochondria play an important role in energy production for the cell. The proper function of a myocardial cell largely depends on the functional capacity of the mitochondria. Therefore it is necessary to establish a novel and reliable method for a non-invasive assessment of mitochondrial function and metabolism in humans. Although originally designed for evaluating myocardial perfusion, ^99m^Tc-MIBI can be also used to evaluate cardiac mitochondrial function. In a clinical study on ischemic heart disease, reverse redistribution of ^99m^Tc-MIBI was evident after direct percutaneous transluminal coronary angioplasty. The presence of increased washout of ^99m^Tc-MIBI was associated with the infarct-related artery and preserved left ventricular function. In non-ischemic cardiomyopathy, an increased washout rate of ^99m^Tc-MIBI, which correlated inversely with left ventricular ejection fraction, was observed in patients with congestive heart failure. Increased ^99m^Tc-MIBI washout was also observed in mitochondrial myopathy, encephalopathy, lactic acidosis and stroke-like episodes (MELAS) and in doxorubicin-induced cardiomyopathy. Noninvasive assessment of cardiac mitochondrial function could be greatly beneficial in monitoring possible cardiotoxic drug use and in the evaluation of cardiac damage in clinical medicine.

## Introduction

Mitochondria play an important role in energy metabolism and are integrally involved in embryonic development, cell signaling activities, cell-cycle control and cell death (1-2). Hence the proper function of a myocardial cell largely depends on the functional capability of the mitochondria. In vitro techniques using isolated mitochondria or cell culture are frequently used to assess the mitochondrial function. However, the techniques have not been fully established for the evaluation of mitochondrial function and metabolism in vivo. It is necessary to establish a novel and reliable method for non-invasive assessment in humans. This article aims to review promising methods for in vivo assessment of mitochondrial function using nuclear cardiology techniques.

### ^99m^Tc-MIBI kinetics in the myocardium

**Figure 1:**
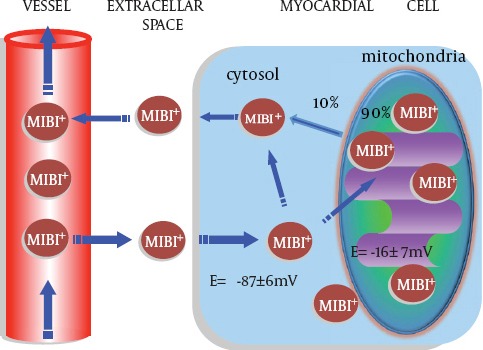
Kinetics of ^99m^Tc-MIBI in the cell. ^99m^Tc-MIBI accumulates and concentrates into cell according to membrane potential

The technetium-99m-labeled agents are used to assess myocardial blood flow. HEXAKIS (2-methoxyisobutylisonitrile) technetium-99m (^99m^Tc-MIBI) is a class of alkylisonitrile technetium compounds designed for noninvasive myocardial perfusion imaging. ^99m^Tc-MIBI concentrates in mitochondria according to membrane potential (3). By using quantitative electron-probe x-ray microanalysis, a strong mitochondrial concentration of ^99m^Tc-MIBI was documented (3). The uptake of ^99m^Tc-MIBI is known to be non-dose dependent. Although originally designed for evaluating myocardial perfusion, ^99m^Tc-MIBI can also be used to evaluate cardiac mitochondrial function. Figure 1 illustrates the kinetics of ^99m^Tc-MIBI in the cell. It does not bind to the cell membrane because no activity was counted with the cell debris resulting from the action of trichloroacetic acid. Piwinica-Worms, Kronauge and Chiu et al provided the fundamental explanation of the mechanism responsible for the cellular accumulation of ^99m^Tc-MIBI (4). Various isonophores and inhibitors that affect the electrical potentials of the plasma and mitochondrial membranes could modify ^99m^Tc-MIBI. The dependence of membrane potential is one of the most important factors in ^99m^Tc-MIBI uptake mechanism. Because the cell membranes are more negatively charged at the mitochondrial level than at the sarcolemmal level, the tracer concentrates preferentially in the mitochondria. The presence of membrane electrical potential drives the accumulation of ^99m^Tc-MIBI. A decrease in mitochondrial function in myocardial cell produces a reduction in the mitochondrial inner matrix potential. The uptake of ^99m^Tc-MIBI seems to be completely driven by a process of passive diffusion. The tracer concentrates preferentially in the mitochondria. At equilibrium, ^99m^Tc-MIBI is sequestered within mitochondria by a large negative potential. An increase in ^99m^Tc-MIBI excretion from cell is thought to be due to P-glycoprotein in tumor cell. Besides the use for a myocardial perfusion tracer, ^99m^Tc-MIBI is known to be a potential functional imaging tracer of multidrug-resistant P-glycoprotein in tumor cells. Increased washout rate of ^99m^Tc-MIBI was correlated with poorer therapy response in some tumors (5).

The consequences of simulated ischemia have been assessed by the experimental study (6). Metabolic change that induces an alteration in the myocardial uptake of ^99m^Tc-MIBI could also alter the trans-membrane potentials, which are accompanied by the myocardial ischemia. Previous experimental study demonstrated that approximately 90% of ^99m^Tc-MIBI in vivo is associated with mitochondria in an energy-dependent manner as a free cationic complex (7). Thus ^99m^Tc-MIBI retention in a mitochondrium relates to mitochondrial function. In addition, Carnonyl cyanide-m-chloro phenylhydrazone (CCCP), a mitochondrial uncoupler, rapidly depletes cellular content of ^99m^Tc-MIBI in the presence of tetraphenylborate (4,8). The results support a potential-dependent mechanism for cell uptake of ^99m^Tc-MIBI (6). In the ischemic model, myocardial ^99m^Tc-MIBI uptake was significantly decreased in mild ischemia, and further decreased in severe ischemia (9). Besides, a lack of ^99m^Tc-MIBI uptake has been reported in some experimental studies (10-11).

The washout rate of ^99m^Tc-MIBI becomes greater in heart failure patients than healthy controls. ^99m^Tc-MIBI washout rate could be used for mitochondrial functional imaging, especially in patients with cardiac dysfunction (12). A recent report showed that an increased washout of ^99m^Tc-MIBI was correlated with a decrease in myocardial mitochondrial mRNA expression or an abnormal morphology of mitochondria in patients with dilated cardiomyopathy (DCM) (13). Several mitochondrial protein mRNA are involved in the myocardial adenosine triphosphate (ATP) production in DCM patients. Mitochondrial ATP production is mainly generated by the tricarboxilic acid cycle in the mitochondrial matrix and the electron transport chain in the mitochondrial membrane (13). Electron microscopic findings revealed that the severity of degeneration in the cristae of mitochondria in the myocardium is correlated with myocardial washout of ^99m^Tc-MIBI (13). Therefore an accelerated ^99m^Tc-MIBI clearance from the myocardium is thought to be related to impaired mitochondrial function, including myocardial damage.

### Ischemic Heart Disease

In a clinical study on ischemic heart disease, Takeishi et al. made a quantitative analysis of ^99m^Tc-MIBI single photon emission computed tomography (SPECT) in acute myocardial infarction patients with successful percutaneous transluminal coronary angioplasty. Reverse redistribution of ^99m^Tc-MIBI was evident after direct percutaneous transluminal coronary angioplasty (14). The presence of increased washout of ^99m^Tc-MIBI was associated with the infarct-related artery and preserved left ventricular function. Fujiwara found the same phenomenon in patients with acute myocardial infarction (15). Both reverse redistribution of ^99m^Tc-MIBI and ^99m^Tc-MIBI /BMIPP mismatch was related to the recovery of left ventricular function, indicating myocardial viability. Reverse redistribution of ^99m^Tc-MIBI may be helpful in prognostic assessment and clinical-decision making in patients with acute myocardial infarction. However, in patients with coronary spastic angina, faster myocardial clearance of ^99m^Tc-MIBI was also observed in the SPECT study, indicating that the ability of myocytes to retain the tracer was impaired due to repetitive brief ischemia by coronary spasm (16). Therefore, the early and delayed ^99m^Tc-MIBI SPECT imaging provides useful information for the diagnosis and responses to the treatment in patients with coronary spastic angina.

### Non-ischemic Cardiomyopathy

**Figure 2:**
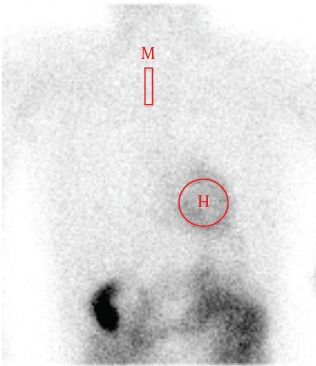
Quantitative analysis by heart-to-mediastinum count ratio on planar image. M = mediastinum; H = heart.

Both dilated and hypertrophic cardiomyopathies are often accompanied by changes in oxidative phosphorylation or respiratory enzyme activities in cardiac tissues (2). In non-ischemic cardiomyopathy, clinical usefulness of using ^99m^Tc-MIBI for cardiac mitochondrial function in patients with cardiomyopathy was recently investigated (12). A 740-MBq dose of ^99m^Tc-MIBI was injected slowly through the antecubital vein and flushed with a 10mL saline solution at rest. The planar and SPECT images were obtained approximately 30 minutes and 3.5 hours after the injection. Cardiac ^99m^Tc-MIBI uptake was measured by heart/mediastinum (H/M) average count ratio, and washout rate was calculated (12). Planar imaging of H/M of ^99m^Tc-MIBI is a simple method that allows comparison of inter-individual and institutional results (Figure 2). An increased washout rate of ^99m^Tc-MIBI was observed in patients with congestive heart failure when compared to the washout rates of those without heart failure (12). Matsuo et al. reported that the washout rate of ^99m^Tc-MIBI was positively correlated with the level of brain natriuretic peptide, end-systolic and end-systolic volume of the left ventricle (12). The washout rate of ^99m^Tc-MIBI was inversely correlated with left ventricular ejection fraction, peak filling rate and first-third ejection fraction. Thus the high ^99m^Tc-MIBI washout rate could be associated with earlier progression of congestive heart failure, suggesting that the evaluation of ^99m^Tc-MIBI washout rate may be useful for predicting the outcomes of congestive heart failure. Since heart failure could be caused by impaired energy production in mitochondria of the cardiomyocyte, the washout rate of ^99m^Tc-MIBI could be a good indicator of myocardial damage or dysfunction even in non-ischemic cardiomyopathy. Besides, the decrease in H/M of iodine-123-metaiodobenzyguanidine (MIBG), a tracer to detect cardiac sympathetic nerve function, was associated with higher washout rate of ^99m^Tc-MIBI in patients with heart failure (11). MIBG scintigraphy and circulating B-type or brain natriuretic peptide (BNP) could be used in combination with perfusion tracer of ^99m^Tc-MIBI to provide complementary information on severity of patients with heart failure (12,17).

### Cardiac Function Evaluation

Quantitative gated single-photon emission computed tomography (QGS) can provide accurate and reproducible values for ejection fraction, regional wall motion, wall thickening and dyssynchrony (18-20). Although a normal heart may contract in a coordinated way, the ischemic burden or myocardial damage results in the inability of cardiomyocyte to produce energy in the mitochondria. Consequently, cardiac dyssynchrony also occurs in such patients. Phase analysis of left ventricular dyssynchrony using a perfusion tracer can be used clinically and gives information on left ventricular dyssynchrony to detect coronary artery disease (21). Furthermore, for evaluating patients with left ventricular bundle branch block or ischemic heart disease, it seems to be more appropriate to evaluate left ventricular wall motion and wall thickening. The assessment of systolic and diastolic function has been commonly used to describe a category of congestive heart failure. These cardiac function evaluations by QGS give useful information on the severity of heart failure.

### Lactic acidosis and stroke-like episodes

Mitochondrial disorders are a heterogeneous group of diseases resulting from abnormalities in mitochondrial deoxyribonucleic acid and function. Cardiac involvement manifesting as hypertrophic (symmetrical or asymmetrical) or dilated cardiomyopathy is frequently observed in mitochondrial myopathy, encephalopathy, lactic acidosis and stroke-like episodes (MELAS) (22). For instance, in a patient with MELAS, increased ^99m^Tc-MIBI washout, which correlated inversely with left ventricular ejection fraction, was observed, whereas increased ^123^I-beta-methyl iodophenyl-pentadecanoic acid (^123^I-BMIPP) uptake was observed in the region of decreased ^99m^Tc-MIBI uptake.

### Doxorubicin-Induced Cardiomyopathy

**Figure 3:**
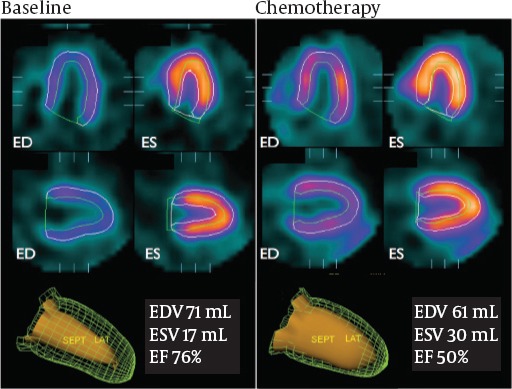
Quantitative gated-SPECT provides cardiac systolic and diastolic functional information. The cardiac function deteriorated in a patient after chemotherapy.

**Figure 4:**
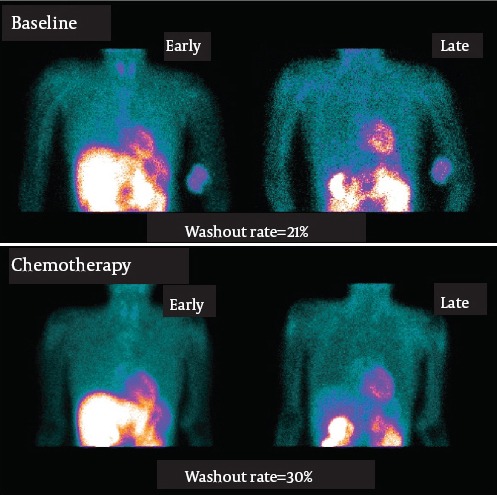
Initial and delayed planar images after administration of ^99m^Tc-MIBI, on baseline and after chemotherapy in a female patient (37 y.o.) with osteosarcoma. The washout rate is increased after chemotherapy (21% on baseline to 30% after chemotherapy).

Doxorubicin (Adriamycin) is one of the original anthracyclines isolated in the early 1960s from the pigment-producing bacterium Streptomyoces percetius, together with daunorubicin. However, doxorubicin has been known to exert many biochemical effects on myocytes, including damage to mitochondria, alteration of nucleic acids and protein synthesis, and lipid peroxidation after free radical generation. Its cardiotoxicity is a dose-dependent process resulting in myocyte damage that may cause congestive heart failure. Monitoring of patients and diagnostic procedures are important because its cardiotoxicity can be lethal. We reported that the ^99m^Tc-MIBI washout rates were higher after the chemotherapy compared to before the chemotherapy in a malignant bone tumor patient with doxorubicin-induced cardiomyopathy (23). Quantitative gated SPECT data showed that the cardiac function decreased in a patient with doxorubicin-induced cardiomyopathy as shown in Figure 3. The washout rate of ^99m^Tc-MIBI increased significantly after the chemotherapy in a patient with doxorubicin-induced cardiomyopathy (Figure 4). ^99m^Tc-MIBI could monitor the change of mitochondrial damage induced by chemotherapy in asymptomatic patients with possible anthracycline antibiotic cardiotoxicity, although there is no difference in SPECT imaging before and after chemotherapy. Moreover, cardiac functions, such as systolic and diastolic function, were impaired more severely in patients with cardiotoxicity than those without (22). Further studies are needed to establish the usefulness of ^99m^Tc-MIBI washout rates in chemotherapy in a larger population of patients (24). Not only doxorubicin but also various kinds of drugs have a potential for cardiotoxicity. The relationship between the increase of myocardial washout of ^99m^Tc-MIBI and poor prognosis in the heart of a patient with cardiotoxicity demonstrate that early detection of these abnormalities and early recognition of myocardial insufficiency is important. Hypertension, ischemia, toxic drugs, or genetic abnormalities cause myocardial damages and may finally result in congestive heart failure. The myocardial washout of ^99m^Tc-MIBI could be a marker for mitochondrial damage in various cardiac diseases.

There may be several factors that affect the values of washout, including age, sex and sympathetic nerve function. The cut-off value between normal and abnormal mitochondrial function is not yet determined. Quantification of the ^99m^Tc-MIBI wash out rate needs to be established by further investigations.

## Conclusion

Noninvasive assessment of cardiac mitochondrial function could be of great benefit in monitoring effective drug use and in the evaluation of cardiac damage in clinical medicine. Although there are a limited number of clinical studies, important features of ^99m^Tc-MIBI outlined in this review suggest utility in assessment of cardiac mitochondrial function. Further studies are needed to fully establish nuclear imaging techniques as a noninvasive assessment of mitochondrial function.
